# Influence of cytochrome P450 and glutathione S transferase polymorphisms on response to nilotinib therapy among chronic myeloidleukemia patients from Pakistan

**DOI:** 10.1186/s12885-022-09605-1

**Published:** 2022-05-08

**Authors:** Samina Naz Mukry, Aneeta Shahni, Uzma Zaidi, Tahir Sultan Shamsi

**Affiliations:** 1 Department of Molecular Biology, National Institute of Blood Diseases & Bone Marrow Transplantation, Karachi, Pakistan; 2 Department of Transplant Immunology and Applied Microbiology, National Institute of Blood Diseases & Bone Marrow Transplantation, Karachi, Pakistan; 3 Department of Post Graduate Studies & Research, National Institute of Blood Diseases & Bone Marrow Transplantation, Karachi, Pakistan; 4 Department of Clinical Hematology, National Institute of Blood Diseases & Bone Marrow Transplantation, Karachi, Pakistan

**Keywords:** Chronic myeloid leukemia, Nilotinib, Treatment, Drug metabolizing enzymes, Response

## Abstract

**Background:**

Cytochrome P450 (CYP) and glutathione S transferases (GSTs) are important biotransforming enzymes responsible for detoxification of anticancer drugs and carcinogens. Polymorphisms in these enzymes may greatly influence the susceptibility to CML and overall efficacy of tyrosine kinase inhibitors. This study was aimed to estimate the possible influence of the polymorphisms of GSTs and CYP in the occurrence of CML as well as in predicting therapeutic outcome of nilotinib therapy in Pakistani CML patients.

**Methods:**

The polymorphic variability in CYP 1A1*2C, GSTP1 (A3131G), GSTT1 and GSTM1 was assessed either by RFLP or multiplex PCR. The BCR ABL1 transcripts were quantified by qPCR to monitor response to nilotinib.

**Results:**

The CYP1A1*2C heterozygous and GSTP1 homozygous polymorphisms seemed to be a contributing factor in developing CML. Altogether, there were 12 non-responders, 66 responders and 21 partial responders. The most frequent genotype was null GSTM1 in responders followed by CYP 1A1 and GSTP1 -wild type (*p* =  < 0.05). Whereas, homozygous GSTP1 and GSTT1 null genotype is significantly higher only among nilotinib non-responders.

**Conclusion:**

Hence, it can be concluded that wild type CYP1A1, GSTP1 and null GSTM1 may be frequently linked to favorable outcome in patients treated with nilotinib as depicted by sustained deep molecular response in most CML patients.

**Supplementary Information:**

The online version contains supplementary material available at 10.1186/s12885-022-09605-1.

## Background

Chronic myeloid leukemia (CML) is a hematological disorder of myeloid progenitor cells. It generally presents with leukocytosis along with accumulation of myelocytes and neutrophils due to uncontrolled over production. The etiology of CML is complex and has been associated with excessive exposure to radiation. The diagnosis of CML is based on the presence or absence of an abnormally translocated chromosome called Philadelphia chromosome(Ph). As a result of this translocation oncogene ABL1 from chromosome 22 moves to the BCR gene on chromosome 9 and BCR gene moves to ABL1position, leading to the formation of BCR/ABL1 kinase. The resultant defective tyrosine kinase stimulates the uncontrolled proliferation of cells resulting in reduced apoptosis causing genomic instability [1, 2]. As a treatment option, targeted inhibitor of tyrosine kinase (TKI) such as imatinib mesylate (Glivec) was used for the treatment of CML. Initially it provided sustained molecular remission in CML patients but up to 33% of patients developed resistance and/or loss of response due to mutations and pharmacokinetic variability. Nilotinib (Tasigna) is a 2^nd^ generation TKI which overcomes the resistance and loss of response issue and achieves good tolerability and response against Ph positive CML [3, 4]. It is an aminophenylpyrimidine derivative with higher selectivity for BCR ABL1 kinase and is approved by the European Medicines Agency (EMA) and the Food and Drug Administration, USA (FDA) as first line treatment for CML due to quicker, deeper and sustained cytogenetic and molecular responses. The associated hepatic and pancreatic toxicities can be monitored through simple tests and are often manageable [5]. While treating leukemia the prime objective is to control or reduce abnormal cell proliferation through targeted TKI therapies. When the adverse events associated with TKIs are beyond the acceptable limits change in TKI is suggested. Unfortunately, the available TKIs to treat CML in Pakistan are limited to imatinib and nilotinib. A high frequency of TKI domain mutation has been reported from local population where switch to nilotinib or ponatinib is suggested as second line [6]. Due to the poor healthcare infrastructure and the unaffordable prolonged cancer treatment nilotinib is being prescribed as first line treatment for chronic and accelerated phase CML in Pakistan with strict monitoring and management of adverse events. Local studies targeting the long term outcome of first line nilotinib treatment and its safety as well as efficacy are required to support such practice.

Further, the risk for adverse drug reactions cannot be overlooked while using second generation TKIs as first line treatment of CML in adults. The interest of many researchers has developed to find out any association of drug resistance and lack of response to TKI in the presence of germline polymorphisms in drug-metabolizing enzymes (DMEs) genes [3] and significant associations of favorable TKI therapeutic outcome (particularly of Imatinib) and polymorphic defects in GST and CYP has been documented [7, 8]. These polymorphisms may regulate drug uptake, metabolic activation and elimination. The DMEs help in xenobiotics deactivation and drug biotransformation. Polymorphisms in these DMEs genes may lead to a loss, reduction or increased activity of these enzymes. Hence, any defect in these genes may result in treatment failures, adverse effects and intoxication [9]. There are three phases of drug metabolism in the human body involving distinct drugs detoxifying enzymes of DME Phase I enzymes such as CYP along with phase II DME help to convert a lipid soluble, non-polar xenobiotic into a polar hydrophilic non- toxic metabolite, which can be readily removed by phase III transporter enzymes [10]. One of the important phase II DME is GSTs which conjugates xenobiotics to water soluble compound such as reduced glutathione (GSH), UDP-glucuronic acid, glycine. Beside conjugation, reduction of hydrogen peroxide resulting in the generation of oxidized glutathione also takes place by the action of GST. GSTs are super family of isozymes which are further divided into eight classes. These are mu (M), theta (T), pi (P), alpha (A), sigma (S), kappa (K), zeta (Z), and omega (O) [11].

The variability in prevalence of DMEs polymorphisms among different population has been widely reported. These differences in metabolic capability due to polymorphic DMEs may impact the drug metabolism and eventually the treatment outcome.The pharmacogenetic screening for DMEs polymorphism may help either in defining personalized dosage of TKI or offering alternate management to non-responsive patients.Due to the dearth of extensive data on the frequencies of polymorphic variations in GSTs and CYP genes in CML patients from Pakistan, this case–control study was conducted with an aim to establish their frequencies to estimate the possible link of these polymorphisms with increased susceptibility to CML and secondly to determine the influence of GSTs and CYP polymorphism in predicting TKI treatment response in CML patients.

## Methods

### Study design

This case control study was conducted at National Institute of Blood Diseases and Bone Marrow Transplantation (NIBD) Karachi, between 2013 to 2019. Informed consent were taken from all patients and healthy subjects after the approval by ethical review committee of NIBD (NIBD/RD/155–37-2013 and conformed to the tenets of the Declaration of Helsinki.

### Study population

A total of 99 patients of CML and 169 age matched healthy controls were enrolled in this study. Patient’s selection was based on the presence of clinical signs and symptoms (abdominal discomfort, fatigue, weight loss and anemia) and Philadelphia chromosome or BCR-ABL gene fusion.

### Treatment and response definitions

In order to study the individual impact of polymorphic defects in DMEs on nilotinib treatment patients receiving nilotinib 300 mg two times/day before 1 h of having meals as first line were enrolled. ELN recommendations 2013 was followed for treatment response [12]. Complete hematological response was defined at the three months of treatment as normal blood count with no immature granulocytes, basophilia or presence of blast along with non-palpable spleen. Major molecular response was defined as transcript ratio of BCR-ABL/ABL less than 0.1%. Deep Molecular response (minor and minimal) was defined as transcript ratio of BCR-ABL/ABL less than 0.01% and 0.0032% on IS respectively. Loss of response at any time after achieving molecular response and failure to achieve molecular response after 12 months were defined as treatment failure. Sokal risk score was also determined. It was defined as a prognostic index for CML patients which predicts response to treatment and survival at diagnosis. The patients were categorized as low, intermediate and high risk having Sokal score < 0.8, 0.8–1.2 and > 1.2 respectively. Responders were those who had achieved deep and major molecular response. Partial responder patients were those who have achieved CHR at 3 months where as Non-responders were those who failed to achieve hematological and molecular response at the given time points. The median follow up time for treatment response assessment was 47 months.

### Molecular analysis for DME polymorphisms

Fresh whole blood samples were collected in EDTA tubes. A peripheral blood smear was prepared for microscopic assessment and cell counting was performed by automated hematolyzerSysmex XN1000 (Kobe, Japan). Genomic DNA was isolated from whole blood using the QIAmp DNA Kit from Qiagen (Qiagen Cat #51,306, USA). The genetic polymorphism analysis for the GSTM1 and GSTT1 genes was based on multiplex PCR approach using previously designed primers by Rocha et al. [13]. The homozygous deletion of GSTT1 and GSTM1 or the null allele results in no expression of these enzymes as confirmed by absence of amplified product by PCR.β-globinwas used as an internal control for each sample. The expected amplicon size was 480 bp in GSTT1 positive individuals and 215 bp in GSTM1. GSTP1A313G and CYP1A1*2C genotype was analyzed by RFLP-PCR as described by Voso et al. [14] and Razmkhak et al. [15].

### Statistical analysis

The frequency of polymorphic DMEs was compared between patient and healthy group by chi square test. The risk rate was also determined along with 95% confidence interval using statistical package SPSS version 22. A *p* value less than 0.05 was considered as statistically significant. Sokal score was determined by Sokal calculator. The OS was calculated by Kaplan–Meier method.

## Results

### Clinical features of CML patients

There were 63 males and 36 females altogether. Furthermore, 97 and 2 were diagnosed with CML in chronic and accelerated phase respectively (Table 1). Out of 99, 66 were responders (male: 36 and female: 30). Six patients expired due to transformation to blast crisis (Table 1). Among the functional biochemical variables only ALP was significantly raised in CML group as compared to healthy subjects (Table 2).

**Table 1:** Clinical features of CML patients.

Clinical Parameters	Number = 99
**Sokal relative risk score**	
< 0.8	66
0.8–1.2	27
> 1.2	6
**Phase at diagnosis**	
Chronic	97
Accelerated	2
Blast Crisis	0
**Treatment Outcome**	
Responders	66
Partial Responders	21
Non-Responders	12
**Hematological Response**	
Complete	66
Partial	30
No	3
**Molecular Response**	
Major MolecularResponse	21
Deep MolecularResponse	45
No Molecular Response	33
**Progression**	
Yes	10
No	89
**Status**	
Alive	93
Dead	6

**Table 2:** Biochemical parameter of CML patients

Biochemistry Parameter	CML Median(interquartile range)	Control Median(interquartile range)	*P* value
Creatinine	1(1.3)	0.7(0.3)	0.073
Urea	17(8.3)	20(9.8)	0.001
**Electrolytes**			
Sodium	140(8.0)	137(5)	0.190
Potassium	4(1.6)	3.6(0.8)	0.028
Chloride	101(5)	102(5)	0.028
Bicarbonates	25(6.0)	24(3)	0.853
**Liver function tests**			
vTotal Bilirubin	1(0.5)	0.7(0.3)	0.001
Direct Bilirubin	0.1(0.6)	0.4(0.2)	0.823
vALP	328(367)	195(75)	0.001
SGPT	22(14.3)	22(47)	0.001

*ALP *Alkaline Phosphatase, *SGPT *Serum Glutamic Pyruvic Transaminase

### Distribution of frequency of CYP1A1*2C, GSTP1A313G, and GSTM1/GSTT1 genotypes

The frequency of CYP1A1*2C, GSTP1A313G, and GSTM1/GSTT1 genotypes in a cohort of 99 CML patients and 169 controls was recorded. The heterozygous genotype of CYP 1A1*2C was more frequent in CML patients than in controls with an OR of 2.6 (*p* = 0.006; Table 3). Similarly, GSTP1 Ile/Val and Val/Val mutant genotype expression was significantly higher in CML patients 20(15%) and 56(38%) compared to control groups 21(12%) and 6(4%) respectively. The GSTT1 and GSTM1 seemed to have no association with occurrence of CML either alone or in combination with each other (Table 3). Further, the impact of combination of multiple polymorphic defects in different DME genes on occurrence of CML was also explored (Supplementary Table 1). Interestingly, seven different combinations listed in Table 3 turned out to be significantly linked to CML with a risk rate of 4.1–11.51.

**Table 3:** Distribution of CYP1A1*2C, GSTP1A313G, and GSTM1/GSTT1 between CML patients and control

Genotype	CML (*n* = 99)	Control (*n* = 169)	*P* value	OR (95% CI)
**CYP1A1 genotype**				
AA	63 (64)	137(81)		
AG	15 (15)	15(9)	**0.006**	2.686(1.326–5.440)
GG	21 (21)	17(10)	0.666	1.235(0.473–3.225)
**GSTP1 genotype**				
Ile/Ile	51 (52)	142(84)		
Ile/Val	36 (36)	6(4)	0.242	1.591(0.731–3.464)
Val/Val	12 (12)	21(12)	**0.001**	0.095(0.031–0.291)
**GSTM1/GSTT1**				
Normal	36 (36.4)	76(46)		
M deletion	36 (36.4)	55(33)	0.747	0.844(0.303–2.357)
T deletion	21 (21.2)	23(13)	0.351	0.611(0.217–1.722)
Double deletion	6 (6)	15(8)	0.147	0.438(0.143–1.338)
**Combined genotype**^**a**^				
AA + Ile/Val	27 (27)	7 (4.2)	**0.001**	8.679(3.613–20.849)
AG + Ile/Val	6 (6)	0	**0.023**	-
AG + M Deletion	3 (3)	0	**0.024**	-
GG + T Deletion	6 (6)	2 (1.2)	**0.042**	5.387(1.066–27.229)
Ile/Val + Normal	12 (12)	2 (1.2)	**0.001**	11.517(2.521–52.618)
Ile/Val + M deletion	12 (12)	5 (3)	**0.006**	4.524(1.544–13.258)
Ile/Val + T deletion	21(12)	4 (2.4)	**0.001**	4.106(3.687–33.452)

^a^ Only statistically significant genotype combinations are included for details see supplementary Table 1

### Association of gene polymorphisms and hematological/molecular response

**Fig. 1:**
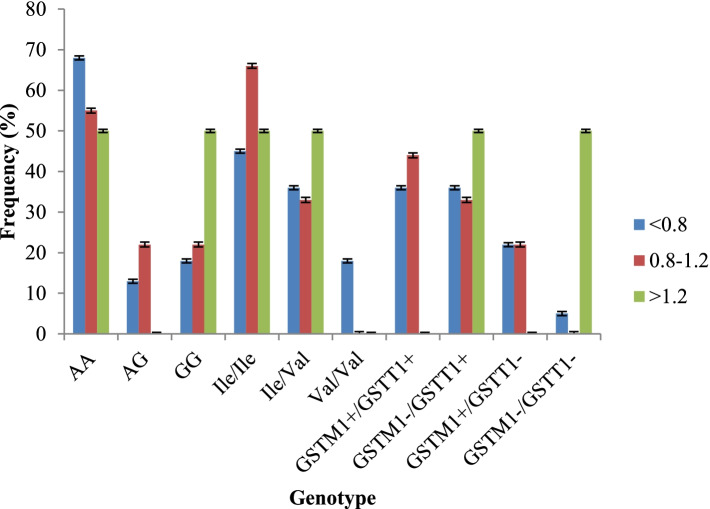
Frequency distribution of DME genotype in different Sokal risk groups

Patients were segregated as per their Sokal score at diagnosis and it was observed that the wild type GSTP1 was significantly associated with low and intermediate risk group patients (Fig. 1). However, the double null deletion (absence of both GSTM1 and GSTT1) had significant association with high risk Sokal relative risk score (Fig. 1). Patients harboring AA wild type of CYP1A1 genotype had higher rate of complete hematological response (42.85%) and deep molecular response (73.33%, Table 4). Similarly, complete hematological response was observed mostly in those patients who carried both GSTM1 and GSTT1 genes. Partial hematological response was noticed to be higher in patients with T deletion (50%) than those who have wild type genes (GSTM1/GSTT1, Table 3). Furthermore, the null GSTM1 was significantly associated with major molecular response (Table 4). Failure to achieve molecular response was also influenced by different gene combinations (supplementary Table 2). It was interesting to note that Val/Val was significantly high in non-responders. A significant association was noted between GSTP1 heterozygous (Ile/Val) genotype and TFS (*p* = 0.005) whereas wild type CYP1A1 and GSTP1 (Ile/ Ile) was frequent in event free survivors (Table 5; *p* = 0.05).

**Table 4:** Association of Hematological and Molecular response with respect to genes polymorphism in CML patients

**Responses**		**Genotype**												
		**CYP1A1**				**GSTP1**				**GSTM1/GSTT1 combined genotype**				
		**AA (a)**	**AG (b)**	**GG (c)**	***P*-value (a *vs* b *vs* c)**	**Ile/Ile (a)**	**Ile/Val (b)**	**Val/Val (c)**	***P*-value (a vs b *vs* c)**	**WT (a)**	**M**^**−**^ **(b)**	**T**^**−**^ **(c)**	**M**^**−**^**/T**^**−**^ **(d)**	***P-*value (a *vs* b *vs* c *vs* d)**
**HR**(*n* = 33)	**CHR** (*n* = 21)	9	9	3	**0.029**	9	12	0	**0.001**	15	6	0	0	**0.001**
	**PHR**(*n* = 12)	9	0	3	0.1	0	3	9	**0.001**	3	3	6	0	**0.001**
**MR** (*n* = 66)	**MMR (*n* = 21)**	12	6	3	0.139	15	6	0	**0.045**	3	9	6	3	**0.045**
	**DMR (*n* = 42)**	33	0	12	**0.001**	27	15	3	0.184	15	18	9	3	0.893
**NMR** (*n* = 33)		18	9	6	0.06	9	15	9	**0.001**	18	9	6	0	**0.032**

*HR* Hematological Response, *MR *Molecular Response, *NMR *No Molecular Response, *CHR *Complete HematologicalResponse, *PHR *Hematologicalresponse, *MMR *Major Molecular Response, *DMR *Deep Molecular Response, *WT *GSTM1 present/GSTT1 present, M^−^ = GSTM1 null/ GSTT1 present, T^−^ = GSTM1present/GSTT1 null, M^−^/T^−^ = GSTM1 null/GSTT1 null

**Table 5:** Impact of different genotypes on over all treatment outcomes

**Treatment outcome**	**Genotype**												
	**CYP1A1**				**GSTP1**				**GSTM1/GSTT1 combined genotype**				
	**AA (a)**	**AG (b)**	**GG (c)**	***P*-value (a *vs* b *vs* c)**	**Ile/Ile (a)**	**Ile/Val (b)**	**Val/Val (c)**	***P*-value (a *vs* b *vs* c)**	**WT** **(a)**	**M **^**−**^** (b)**	**T **^**–**^ **(c)**	**M **^**−**^**/T**^**−**^ **(d)**	***P*-value (a *vs* b *vs* c *vs* d)**
**Responders (*n* = 66)**	45	6	15	**0.05**	42	21	3	**0.001**	18	27	15	6	**0.032**
**Partial Responders (*n* = 21)**	9	9	3	**0.001**	9	12	0	**0.034**	15	6	0	0	**0.001**
**Non-Responders (*n* = 12)**	9	0	3	0.295	0	3	9	**0.001**	3	3	6	0	0.068

### Impact of DME genotypes on over all treatment outcome

**Fig. 2:**
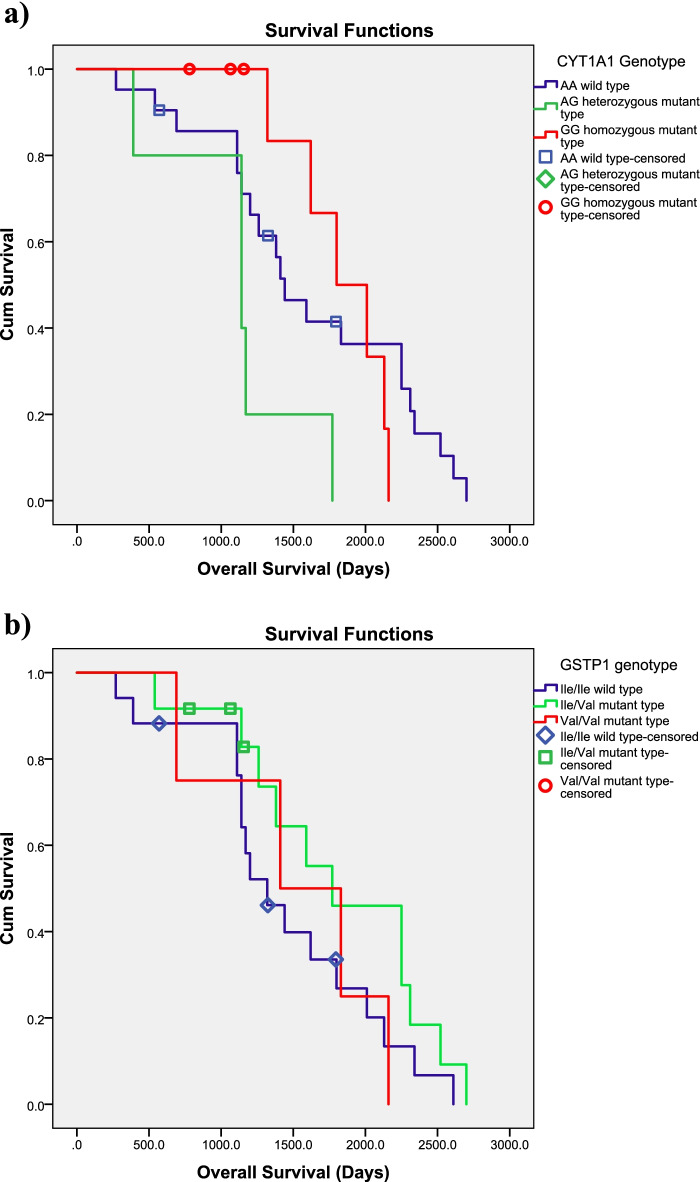
Kaplan–Meier survival curves for the overall survival (Days) after TKI treatment until death as an endpoint is represented on the basis of DME genotype. **a** CYP1A1 genotype (log rank test *P* = 0.05). **b** GSTP1A313G (log rank test *P* value: 0.029)

The overall outcome of treatment was also determined at mean follow up of 51.33 months. There were 66 responders, 21 partial responders and 12 non-responders to nilotinib treatment (Table 5). It was interesting to note that the wild type CYP1A1, GSTP1 and GSTM1 deletion was significantly frequent in responders. The partial responders carried heterozygous mutant genotypes of CYP1A1, GSTP1 and wild type of GSTM1/GSTT1 whereas homozygous GSTP1genotype was significantly linked to treatment failure. The GSTT1 deletion was also frequent in failure group but it could not reach the statistical significance. Out of 99 patients 6 patients died despite treatment and irrespective of their Sokal risk score. All these patients were non-responders male with an average age of 41 years. The relationship between DMEs polymorphisms and overall survival of nilotinib treated patients was also studied by log rank test. The GSTM1 and GSTT1 polymorphisms did not affect the overall survival. Whereas, the CYP1A1 AA genotype is associated with better survival than the GG homozygous genotype (log rank test *P* = 0.05). Furthermore, GSTP1A313G heterozygous mutant genotype tends to influence better survival (log rank test *P* value: 0.029, Fig. 2).

## Discussion

The genetic alterations such as mutations and SNPs in many regulatory genes including TP53, KRAS, DDR2, KLK3 etc. are the leading cause of increased susceptibility to cancer. The variation in these regulatory genes influence regulation of vital cellular processes such as cell cycle, cell differentiation/ proliferation, DNA synthesis/repair, apoptosis, breakdown or synthesis of exogenous and endogenous substances and immune response.There are several different chemotherapeutic agents used to treat cancer by destroying rapidly growing cancer cells at almost every step of cell cycle. The DMEs play key role in the activation and/or detoxification of these drugs via biotransformation [16, 17]. The presence of polymorphic defects in DME’s has been confirmed as a contributing factor in occurrence and pathogenesis of leukemia and other cancers from different ethnicities. To the best of our knowledge, this is the first case control study encompassing influence of polymorphic defects in DMEs on the CML susceptibility and nilotinib treatment outcome from Pakistan. Previous reports on absence of association of polymorphic defects of DMEs and occurrence of CML are abundant [18–20]. Only few researchers so far have documented association of DMEs with risk of CML [21]. One possible reason for this variance in observations might be variable degree of either exposure to harmful environmental factors or difference in frequency of polymorphic defects in DMEs among different populations causing accumulation of these. The germline gene polymorphism CYP1A1*2C in first phase DME results in gain of function due to overproduction of enzyme. Presence of excessive CYP1A1 results in decreased bioavailability of therapeutics and increased accumulation of harmful drug metabolic byproducts leading to more drug adverse events [22]. In the current study, healthy individuals with the heterozygous genotype (AG) of the first phase DME CYP 1A1 were 2.6 times more susceptible to CML. Similarly, Hadeil et al., also reported a high prevalence of AG in 200 CML patients (OR: 18.38) [23]. The homozygous deletion of GSTM1 and GSTT1 genes (null allele) results in no expression of these enzymes. About 30–60% individuals have GSTT1 null allele [24]. Inconsistent results for presence of GSTT1 and GSTM1 null genotype and occurrence of CML were reported in the past from different populations. Hishida et al., documented a little impact of GSTT1 null allele with CML whereas among Argentineans both GSTM1 and GSTT1 null genotype were not a contributing factor in increased susceptibility to CML in healthy individuals [25]. Similarly, homozygous deletion of both these genes seemed to have no influence on CML pathogenesis during the present study (Table 2). On the contrary a protective effect of GSTT1 was reported by Bhat et al. from Kashmiris [26]. Converse to the results of Rostami et al. the GSTP1 heterozygous genotype (Ile/Val) was frequent in healthy individuals and did not confer any risk of CML alone [27]. But when in combination with other DME genotypes it seemed to contribute more in pathogenesis of CML with relatively high CML chance (Table 2).

In case of CML the abnormal kinase activity of BCR ABL1 enzyme is controlled by treatment with TKIs. Imatinib was the first TKI successfully used to treat CML. Despite promising results; an increased rate of resistance (in ~ 30% patients) due to inconsistent pharmacokinetics, genetic variability in TKI binding sites and associated toxicities nilotinib was introduced as a second line TKI with a separate binding site. As per current ELAN recommendation nilotinib can be used as first line drug. Serious side effects of most TKIs have been linked to metabolic activation of these TKIs by cytochrome P450 mediated biotransformation. The nilotinib is considered safer than imatinib and is greatly metabolized in liver by the action of CYP3A. The extra hepatic metabolism of nilotinib if any is poorly understood. Presence of wild type -Cyp1A1, -GSTP1 and GSTM1 null genotype seemed to be significantly linked to a more sustained DMR whereas patients harboring GSTP1 Ile/Val and wild type GSTM1/GSTT1 failed to achieve any molecular response (*p* < 0.05;Table 3).

Only few studies have shown the impact of DMEs polymorphisms on treatment outcome in CML. Altogether, 6 patients failed to respond to nilotinib and expired. All these patients had defects in DMEs genes and also harbored T315I kinase domain mutation already associated with nilotinib non-responsiveness in CML patients [6]. Hence it can be assumed that the sudden death in these patients may also be associated with insufficient bio-detoxification due to defective DMEs.

In Malaysians the polymorphic GSTP1 genotypes and null GSTT1 was linked with imatinib resistance similarly these genotypes were frequent in partial and non-responder to nilotinib during the current study [28]. The frequency of GSTM1 null genotype was significantly linked to good response in nilotinib treated patients (Table 5). Moreover, the frequency was also high in patient’s achieving MMR (42.85%) and DMR (40%) but it could not reach the statistical significance. While studying the copy number variation analysis (CNV) of cytochromes and GSTs to predict efficacy of TKI in CML the researchers proposed that GSTM1 may not be a specific marker to prognose treatment outcome but GSTT1 may be associated with CML [3]. Hence, null GSTM1 seemed to be frequent in nilotinib responders with possible influence on drug response..This is contrary to previous study where the GSTM1 null allele was associated with treatment failure in imatinib treated CML patients [29]. The overproduction or absence of active enzyme may have direct or indirect influence on drug response due to increased or diminished drug metabolism and may be used drug dosage designing.

## Conclusion

This study concluded that heterozygous mutant of CYP1A1 and homozygous mutant of GSTP1 gene might be a contributing factor in CML pathogenesis. This study also shows that polymorphic variation in CYP1A1, GSTP1 and GSTM1 null genotype might influence the molecular response and treatment outcome in CML patients. Further studies at larger scale should be done to evaluate the impact of polymorphic DME genes on treatment related toxicity (if any) and relapse of disease.

## Supplementary Information

**Additional file 1: Supplementary Table 1.** Impact of combination of multiple polymorphic defects in different DME genes on occurrence of CML.

## Data Availability

The data produced during the present study will be available on request from the corresponding author.

## Abbreviations

A: Alpha
CML: Chronic Myeloid Leukemia
DMEs: Drug-Metabolizing Enzymes
GSH: Glutathione
GST: Glutathione S transeferase
K: Kappa
M: Mu
O: Omega
P: Pi
Ph: Philadelphia chromosome
S: Sigma
T: Theta
TKI: Tyrosine Kinase Inhibitor
UV: Ultraviolet
Z: Zeta
