# Supplementary material for: Influence of cytochrome P450 and glutathione S transferase polymorphisms on response to nilotinib therapy among chronic myeloidleukemia patients from Pakistan
Source: BMC Cancer. 2022 May 8;22:519. doi: 10.1186/s12885-022-09605-1 (PMC9080200; doi:10.1186/s12885-022-09605-1)
Supplement: Supplementary file 1 — Additional file 1: Supplementary Table 1. Impact of combination of multiple polymorphic defects in different DME genes on occurrence of CML. [file 12885_2022_9605_MOESM1_ESM.docx]

**Supplementary Table 1: Impact of combination of multiple polymorphic defects in different DME genes on occurrence of CML**

|  | Combined mutations | CML | Control | P value |
| --- | --- | --- | --- | --- |
| 1 | AA+ILE/ILE | 27 | 151 | 0.001 |
| 2 | AA+ILE/VAL | 27 | 7 | 0.001 |
| 3 | AA+VAL/VAL | 9 | 29 | 0.068 |
| 4 | AG+ILE/ILE | 9 | 21 | 0.403 |
| 5 | AG+ILE/VAL | 6 | 0 | 0.001 |
| 6 | AG+VAL/VAL | 0 | 7 | 0.040 |
| 7 | GG+ILE/ILE | 15 | 22 | 0.625 |
| 8 | GG+ILE/VAL | 3 | 4 | 0.742 |
| 9 | GG+VAL/VAL | 3 | 4 | 0.742 |
| 10 | AA+GSTM1+/GSTT1+ | 18 | 104 | 0.001 |
| 11 | AA+GSTM1+/GSTT1- | 27 | 69 | 0.025 |
| 12 | AA+GSTM1-/GSTT1+ | 12 | 42 | 0.012 |
| 13 | AA+GSTM1-/GSTT1- | 6 | 17 | 0.259 |
| 14 | AG+GSTM1+/GSTT1+ | 12 | 10 | 0.074 |
| 15 | AG+GSTM1+/GSTT1- | 0 | 13 | 0.005 |
| 16 | AG+GSTM1-/GSTT1+ | 3 | 0 | 0.023 |
| 17 | AG+GSTM1-/GSTT1- | 0 | 6 | 0.058 |
| 18 | GG+GSTM1+/GSTT1+ | 6 | 10 | 0.962 |
| 19 | GG+GSTM1+/GSTT1- | 9 | 14 | 0.820 |
| 20 | GG+GSTM1-/GSTT1+ | 6 | 2 | 0.024 |
| 21 | GG+GSTM1-/GSTT1- | 0 | 6 | 0.058 |
| 22 | ILE/ILE +GSTM1+/GSTT1+ | 21 | 102 | 0.001 |
| 23 | ILE/ILE +GSTM1+/GSTT1- | 18 | 74 | 0.001 |
| 24 | ILE/ILE +GSTM1-/GSTT1+ | 9 | 38 | 0.005 |
| 25 | ILE/ILE +GSTM1-/GSTT1- | 3 | 15 | 0.065 |
| 26 | ILE/VAL+GSTM1+/GSTT1+ | 12 | 2 | 0.001 |
| 27 | ILE/VAL +GSTM1+/GSTT1- | 12 | 5 | 0.003 |
| 28 | ILE/VAL +GSTM1-/GSTT1+ | 21 | 4 | 0.001 |
| 29 | ILE/VAL +GSTM1-/GSTT1- | 3 | 2 | 0.281 |
| 30 | VAL/VAL+GSTM1+/GSTT1+ | 3 | 16 | 0.048 |
| 31 | VAL/VAL +GSTM1+/GSTT1- | 6 | 9 | 0.800 |
| 32 | VAL/VAL +GSTM1-/GSTT1+ | 3 | 4 | 0.742 |
| 33 | VAL/VAL +GSTM1-/GSTT1- | 0 | 12 | 0.007 |

**Supplementary Table 2: Association of Molecular response with respect to combined mutations genes polymorphism in CML patients**

|  | Combined mutations | Major molecular response | Deep molecular response | failure | P value |
| --- | --- | --- | --- | --- | --- |
| 1 | AA+ILE/ILE | 9 | 15 | 3 | 0.012 |
| 2 | AA+ILE/VAL | 3 | 15 | 9 | 0.270 |
| 3 | AA+VAL/VAL | 0 | 3 | 6 | 0.05 |
| 4 | AG+ILE/ILE | 3 | 0 | 6 | 0.014 |
| 5 | AG+ILE/VAL | 3 | 0 | 3 | 0.052 |
| 6 | AG+VAL/VAL | 0 | 0 | 0 | - |
| 7 | GG+ILE/ILE | 3 | 12 | 0 | 0.005 |
| 8 | GG+ILE/VAL | 0 | 0 | 3 | 0.045 |
| 9 | GG+VAL/VAL | 0 | 0 | 3 | 0.045 |
| 10 | AA+GSTM1+/GSTT1+ | 0 | 12 | 6 | 0.033 |
| 11 | AA+GSTM1+/GSTT1- | 6 | 12 | 9 | 0.987 |
| 12 | AA+GSTM1-/GSTT1+ | 3 | 6 | 3 | 0.803 |
| 13 | AA+GSTM1-/GSTT1- | 3 | 3 | 0 | 0.098 |
| 14 | AG+GSTM1+/GSTT1+ | 3 | 0 | 9 | 0.001 |
| 15 | AG+GSTM1+/GSTT1- | 0 | 0 | 0 | - |
| 16 | AG+GSTM1-/GSTT1+ | 3 | 0 | 0 | 0.003 |
| 17 | AG+GSTM1-/GSTT1- | 0 | 0 | 0 | - |
| 18 | GG+GSTM1+/GSTT1+ | 0 | 3 | 3 | 0.384 |
| 19 | GG+GSTM1+/GSTT1- | 3 | 6 | 0 | 0.084 |
| 20 | GG+GSTM1-/GSTT1+ | 0 | 3 | 3 | 0.384 |
| 21 | GG+GSTM1-/GSTT1- | 0 | 0 | 0 | - |
| 22 | ILE/ILE +GSTM1+/GSTT1+ | 3 | 12 | 6 | 0.453 |
| 23 | ILE/ILE +GSTM1+/GSTT1- | 6 | 9 | 3 | 0.178 |
| 24 | ILE/ILE +GSTM1-/GSTT1+ | 3 | 6 | 0 | 0.084 |
| 25 | ILE/ILE +GSTM1-/GSTT1- | 3 | 0 | 0 | 0.003 |
| 26 | ILE/VAL+GSTM1+/GSTT1+ | 0 | 3 | 9 | 0.004 |
| 27 | ILE/VAL +GSTM1+/GSTT1- | 3 | 6 | 3 | 0.803 |
| 28 | ILE/VAL +GSTM1-/GSTT1+ | 6 | 9 | 6 | 0.637 |
| 29 | ILE/VAL +GSTM1-/GSTT1- | 0 | 3 | 0 | 0.156 |
| 30 | VAL/VAL+GSTM1+/GSTT1+ | 0 | 0 | 3 | 0.045 |
| 31 | VAL/VAL +GSTM1+/GSTT1- | 0 | 3 | 3 | 0.384 |
| 32 | VAL/VAL +GSTM1-/GSTT1+ | 0 | 0 | 3 | 0.045 |
| 33 | VAL/VAL +GSTM1-/GSTT1- | 0 | 0 | 0 | - |
